# Effect of contacts with health professionals on modern contraceptives uptake during the first 6 weeks after child birth: a prospective cohort study in Arsi Zone

**DOI:** 10.1186/s40834-023-00237-9

**Published:** 2023-07-19

**Authors:** Gebi Husein Jima, Jelle Stekelenburg, Hailu Fekadu, Tegbar Yigzaw Sendekie, Regien Biesma

**Affiliations:** 1 Department of Public Health, College of Health Science, Arsi University, Asella, Ethiopia; 2 Department of Health Sciences, Global Health Unit, University Medical Centre Groningen, Groningen, the Netherlands; 3 Department of Obstetrics and Gynaecology, Medical Centre Leeuwarden, Leeuwarden, the Netherlands; 4 Jhpiego, Addis Ababa, Ethiopia

**Keywords:** Postnatal contraceptives, Women, Inter-pregnancy interval, Follow-up, Ethiopia

## Abstract

**Background:**

Healthy timing and spacing of pregnancy refers to the spacing between deliveries and subsequent pregnancies. The World Health Organization recommends waiting at least 24 months between the date of the live birth and the conception of the subsequent pregnancy in order to lower the risk of unfavorable maternal, perinatal, and newborn outcomes. Low use of contraception contributes to the high level of short inter-pregnancy intervals. Different studies conclusively demonstrate that this is a reality existing in Ethiopia right now. Limited data is available regarding the effects of contacts with health professionals on the use of contraception during the postnatal period.

**Methods:**

A prospective cohort study was performed from October 01, 2020 to March 01, 2021. The study included 418 postnatal women who gave birth during the previous week. They were followed throughout the full postnatal period. A pre-tested structured questionnaire was used to gather the data. Data were gathered twice: once during the first week following birth and once again from the eighth to the 42nd day after birth. Epi-Info version 7 was used to enter data, which was subsequently exported to SPSS version 21 for analysis. The effect of contacts with health professionals where contraceptives were discussed on contraception uptake was measured using adjusted relative risk and its 95% confidence interval.

**Results:**

Modern contraceptive uptake rate during the postnatal period was 16% (95% CI: 12.50-19.50%). Contraceptive use was 3.56 times more likely in women who were counseled about contraceptives during a contacts with health professionals at a health facility compared to those who did not have a contact (aRR = 3.56, 95% CI: 1.97–6.32). Women’s age, place of residence, knowledge of whether they can become pregnant before menses return, menses return after birth, and resuming sexual activity after birth were all significantly associated with contraceptive use during the first six weeks following child birth.

**Conclusions:**

Modern contraceptive uptake rate during the postnatal period among women in the study area was low. Contacts with health professionals where contraception is discussed was the main factor associated with contraception uptake during the postnatal period. We recommend that the Arsi Zone Health Office, the Weardas Health Office in the Arsi Zone, and the health care providers in the Arsi Zone health facilities strengthen contraceptive counseling in postnatal health services to reduce the proportion of women with short inter-pregnancy intervals.

## Introduction

Healthy timing and spacing of pregnancy refer to the spacing between deliveries and subsequent pregnancies [1]. In order to reduce the risk of adverse maternal and perinatal outcomes, the World Health Organization (WHO) suggests waiting at least 24 months between the date of the live birth and the conception of the next pregnancy [2, 3]. Low use of contraception contributes to the high level of short inter-pregnancy intervals [2].

The resting time between pregnancies allows the mother time to recover from pregnancy, labor, and lactation as well as time to replenish her nutritional reserves, including calcium, iron, and vitamins. It also allows her uterus to return to its natural state and gives the lastborn child time to establish his or her right to comprehensive care and sufficient breastfeeding [1].

Sub-Saharan Africa (SSA) is the region with the highest total fertility rate (an average of 5.5 children born to a woman during her lifetime); the highest population growth rate (2.4%); and the lowest contraceptive use rate (23%). These data are caused by pregnancies that are too early, too many, or spaced too closely. Only approximately 5% of women who have just given birth want another pregnancy within two years [4]. Many women are unable to access contraceptive services soon after giving birth, despite their need to space pregnancies in a healthy way. The deficiency of appropriate and consistent messaging at critical service points in the continuum of care is a significant contributor to this gap. This in turn contributes to high fertility rates and poor health for both the mothers and the children [3, 4].

Short inter-pregnancy intervals have been linked to adverse maternal and infant health outcomes. [5, 6] .

More recent research has shown [7, 8] that there are higher risks for adverse perinatal and maternal outcomes when there is a short inter-pregnancy interval of less than 18 months.

These include perinatal and neonatal complications such as preterm delivery [5, 8], low birth weight, small for gestational age [5, 8], birth abnormalities [9] and autism [10], as well as maternal complications like uterine rupture in women who underwent caesarean section in the previous birth [11, 12].

The Maternal Depletion Syndrome hypothesizes that mothers with short inter-pregnancy intervals frequently do not have enough time to replenish macro- and micro-nutrients, which may result in the mother and fetus competing for nutrients [13]. An interval of 18 to 24 months has been associated with a lower relative risk of these perinatal and maternal outcomes [6].

In Ethiopia, the rate of married women utilizing modern contraception has significantly increased over the past two decades, from 6% to 2000 to 41% in 2019 [14]. As a result, the total fertility rate declined from 7.2 births per woman in 1990 to 4.1 in 2019 [15]. However, there is still a gap between the number of women who want to prevent or delay pregnancy and the number who use modern contraception. A quarter of currently married women have an unmet need for family planning services; 16% do not meet the two-year birth spacing requirement, and 9% do not meet the birth limitation requirement [16].

Although the Ethiopian Federal Ministry of Health (MOH) implementation plan calls for increasing the use of contraceptives and addressing the unmet need for family planning, the practice gap indicates that all levels of health facilities should place a higher priority on addressing the contraceptive needs of postpartum women [16]. Counseling about contraceptives is not given priority in postpartum health services in Ethiopia. One crucial strategy is to provide women with contraception soon after giving birth in a facility [7, 8].

Few studies have been done in some regions of Ethiopia on the impact of contacts with health professionals at a health facility on the adoption of contraception throughout the extended postpartum period, or the first year after giving birth. However, there are no data about how contacts with health professionals at a health facility affect contraceptive use in Ethiopia during the postnatal period. Therefore, this study assessed the effects of contacts with health professionals at a health facility where contraceptives are discussed on the adoption of modern contraceptives in the first six weeks following childbirth.

## Materials and methods

### Study setting

This study was conducted in Arsi Zone, Oromia Regional State, Ethiopia, from October 1, 2020 to March 1, 2021. Arsi Zone is one of the zones in Oromia National Regional State. Asella, the capital of the Arsi Zone, is located 175 kilometers from Addis Ababa, the nation’s capital city, in the southeast direction. The Zone encompasses 19,825.22 km2 and is divided into 26 districts [17, 18]. In the middle of 2022, the population was officially estimated at 3,894,248. Astronomically, the zone lies between 7008’58’’N − 8049’00’’N latitude and 38,041’55’’E − 40,043’56’’E longitude [19].

### Study design

We used a prospective cohort study to assess the effect of contacts with health professionals at a health facility on the uptake of modern contraceptives during the first 6 weeks after childbirth.

### Study population

Postnatal women who gave birth within the last 7 days in the selected districts were enrolled in the study and followed prospectively for the entire postnatal period (6 consecutive weeks). In addition to women who gave birth at home, women who gave birth in the health facilities were also included in the study before they were discharged from the health facility. In our study, exposure was defined as contact with health professionals at the facility. Hence, women who had contacts with health professionals at a health facility were in exposed groups, and women who didn’t have contacts with a health professional at a health facility were in non-exposed groups.

### Inclusion criteria

Postnatal women who gave birth within the last seven days and residents of the selected districts were included.

### Study variables

#### Outcome variable

Modern contraceptive uptake occurs during the postnatal period (the first 6 weeks after childbirth).

#### Independent variables

Contacts with health professionals at a health facility where contraceptives are counseled was the primary independent variables considered in this study. Moreover, the mere women’s contacts with health professionals at a health facility, women’s age, women’s place of residence, women’s and their husbands’ educational levels, knowledge of whether they can become pregnant before menses return, menses returning after childbirth, sexual activity beginning after childbirth, desire to have more children, desire to wait some time before the next pregnancy, and women’s knowledge of the recommended Healthy Timing and Spacing of Pregnancy (HTSP) were also considered independent variables.

### Sample size and sampling process

A sample size was estimated to detect a 10% difference in contraceptive acceptance rate (CAR) between those who had and didn’t have contacts with health professionals at a health facility with a 95% confidence level, a 5% margin of error, a 1:1 unexposed to exposed ratio, 80% statistical power, and a 10% loss to follow-up. The assumption of a 10% CAR was based on data on early postpartum family planning adoption from a previously conducted study [20]. Consequently, a target sample of 432 postpartum mothers was generated.

Six districts (Tiyo, Hetosa, Lode Hetosa, Zeway Dugda, Digelu Tijo, and Dodota) were randomly selected from the 26 districts in the Arsi zone. Five kebeles (four rural and one urban) were selected at random from each of the districts that were chosen, totaling thirty kebeles (24 rural and 6 urban) for the study. Then, all the women from each kebele who met the criteria for inclusion were included. Eligible study participants were identified from the list of postnatal women at health posts in the selected kebeles. If a list was not up-to-date, health extension workers helped identify eligible women in the village. The interviewed women were also asked if they knew other postnatal women in their village.

### Data collection tools and procedures

The questionnaires were prepared in English and translated into local languages (Afan Oromo and Amharic). It comprises items related to socio-economic and socio-demographic characteristics, reproductive related characteristics, contacts with health professionals at a health facility, and contraceptive uptake during the postanatal period after birth. The questionnaire was pre-tested on 5% of the total sample in Munessa district.

For data collection, ten experienced healthcare professionals fluent in Afan Oromo and Amharic were recruited. Six public health specialists were also hired to supervise the field data collection. Data collectors and supervisors received three days of training on the data collection tools and procedures. Each district’s field supervisor was tasked with supervising the quality of data collection through observation of interviews and verification of completed questionnaires.

At enrolment (during the first week following the current birth), all study participants’ socio-demographic and other basic information was gathered. During the second survey, a visit to the women’s home was made using their complete contact information that was captured at the enrollment. Within a week of the 42nd day following the current delivery, each study participant was contacted to collect information pertaining to the time frame between the 8th and 42nd days following the current birth. The required information, such as the number of contacts with health professionals at a health facility where women received information about contraceptives and the uptake of those contraceptives, was gathered during the follow-ups. Contact with a health professional after a woman began taking contraception was not included.

### Data processing and analysis

Epi-Info version 7 was used to enter the data, which was then exported to SPSS version 21 for analysis. The data was checked for consistency and completeness before analysis. Descriptive statistics were used to describe the sample as per the considered characteristics. The proportion of postnatal women who use modern contraception was determined and compared for those who had and did not have interaction with a health professional at a health facility. Bivariate analysis was carried out to select candidate variables for the final model at p-values below 0.2. An adjusted relative risk (aRR) was generated for each variable, and the independence of any association was controlled by entering all variables into the model using the backward stepwise method (backward conditional method). Adjusted relative risk (aRR) and its 95% confidence interval were used to calculate the strength of the association between contraceptive use during the first six weeks following the current birth and the health-care contacts where family planning was discussed. P-values under 0.05 were regarded as statistically significant.

## Results

### Socio-demographic characteristics of study participants

Four hundred eighteen postnatal women who gave birth within the preceding week were included in the follow-up, which is 3.3% less than the calculated sample. Women who had and did not have contacts with health professionals at a health facility were slightly different in most of the socio-demographic characteristics (See Table 1).

**Table 1:** Socio-demographic characteristics and comparison between postnatal women who had and did not have contacts with health professionals at a health facility in the Arsi Zone, 2022

Variables	Had contact with health professionals (N = 226) N(%)	No contacts with health professionals (N = 192) N(%)	P-value
Districts			
Hetosa	30(13.3)	34(17.7)	0.007*
Lode Hetosa	51(22.6)	11(5.7)	0.073
Digelu Tijo	12(5.3)	53(27.6)	0.001*
Ziway Dugda	66(29.2)	7(3.6)	0.002*
Dodota	9(4.0)	61(31.8)	0.001*
Tiyo	58(25.7)	26(13.5)	0.001*
Age			
< 20	40(17.7)	29(15.1)	0.187
20–29	131(58)	119(62)	0.412
>=30	55(24.3)	44(22.9)	0.756
Woman’s current marital status			
Never Married	9(4.0)	8(4.2)	0.808
Currently Married	216(95.6)	182(94.8)	0.914
Widowed	0(0.0)	2(1.0)	0.999
Divorced	1(0.4)	0(0.0)	1.000
Woman’s religion			
Muslim	170(75.2)	130(67.7)	0.159
Catholic	3(1.3)	3(1.6)	0.941
Protestant	6(2.7)	9(4.7)	0.543
Orthodox	47(20.8)	50(26.0)	0.761
Woman’s Ethnicity			
Oromo	209(92.5)	182(94.8)	0.494
Amhara	14(6.2)	7(3.6)	0.866
Others	3(1.3)	3(1.6)	0.460
Woman’s Residence			
Rural	171(75.7)	123(64.1)	0.010*
Urban	55(24.3)	69(35.9)	0.210
Woman’s Education level			
Cannot Read and Write	37(16.4)	15(7.8)	0.641
Can Read and Write	11(4.9)	30(15.6)	0.001*
Primary education	133(58.8)	125(65.1)	0.023*
Secondary and above	45(19.9)	22(11.5)	0.006*
Husband’s Education level			
Cannot Read and Write	12(5.3)	5(2.6)	0.100
Can Read and Write	30(13.3)	25(13)	0.246
Primary education	97(42.9)	137(71.4)	0.026*
Secondary and above	87(38.5)	25(13)	0.521
Household sources of income			
Farming	162(71.7)	136(70.8)	0.903
Trade	21(9.3)	26(13.5)	0.303
Employed in Government or Private	17(7.5)	9(4.7)	0.404
Other	26(11.5)	21(10.9)	0.467

### Reproductive characteristics of study participants

Forty-four (65.7%) of those who used contraception and 39 (11.1%) of those who did not report that their menses had already returned. Fifty-nine (88.1%) of contraceptive users and 128 (36.5%) of non-users had already resumed sexual intercourse in the first 42 days after birth. Sixty-three (94%) of contraceptive users and 186 (53%) of non-users believed that a woman could get pregnant before her menstrual cycle resumed (Table 2).

**Table 2:** Reproductive characteristics of postnatal women in Arsi Zone, 2022

Variables	Modern contraceptive users ( N = 67) N1(%)	Non-Users ( N = 351) N1(%)	P-value
Menses return			
Yes	44(65.7)	39(11.1)	0.001*
Can get Pregnant before menses returns			
Yes	63(94)	186(53)	0.001*
Sexual intercourse begun			
Yes	59(88.1)	128(36.5)	0.001*
Period abstained from sexual intercourse			
≤ a month	49(73.1)	256(72.9)	0.973
>a month	18(26.9)	95(27.1)	
Desire to have more children			
Yes	65(97)	306(87.2)	0.033*
Want to wait some time before the next pregnancy			
Yes	62(92.5)	288(82.1)	0.040*
Want to delay the next pregnancy by:			
<2 years	5(7.5)	74(21.1)	0.013*
≥2 years	62(92.5)	277(78.9)	0.000*
Respondents who understand the risk of closely spaced pregnancies			
Yes	64(95.5)	328(93.4)	0.522
Healthy timing and spacing of Pregnancy			
< 18 months	4(6.0)	126(35.9)	0.001*
18 months-24 months	25(37.3)	144(41.0)	0.001*
> 24 months	38(56.7)	81(23.1)	

### Contacts with health professionals at a Health facility

One hundred ninety (45.5%) and 226 (54.1%) women had contacts with health professionals at a health facility in the first 7 days and the eighth to 42nd days following the current birth, respectively. Health centers were the most frequent place for these contacts during the first (82.6%) and last visits (48.2%). Health post was the second most common point of contact in the last visit (Table 3).

**Table 3:** Postnatal women’s contacts with health professionals at a Health facility and modern contraceptive use in Arsi Zone, 2022

Variables	1st Visit N1 (%)	Last Visit N2 (%)
Contacts with health professionals for MNCH service (N = 418)		
Yes	190(45.5)	226(54.1)
No	228(54.5)	192(45.9)
MNCH service received (N1 = 190, N2 = 226)		
Woman/baby check	146(76.8)	84(37.2)
Immunization	37(19.5)	128(56.6)
Other	7(3.7)	14(6.2)
Place for the first check (N1 = 190, N2 = 226)		
Hospital	11(5.8)	4(1.8)
Health center	157(82.6)	109(48.2)
Private clinic	6(3.2)	17(7.5)
Health post	5(2.6)	96(42.5)
Home visit	11(5.8)	-
Contraceptive initiated (N1 = 190, N2 = 226)		
Yes	37(19.5)	75(33.2)
No	153(80.5)	151(66.8)
Timing of contraceptive initiated(N1 = 190, N2 = 226)		
Birth to 3rd day	20(10.5)	NA
4th day to 7th day	17(8.9)	NA
8th to 42nd days	NA	33(14.6)
After 42nd days	NA	42(18.6)

**Key** N1 is the number of women who had contacts with health professionals during their first visit, and N2 is the number of women who had contacts with health professionals during their second visit

**Fig. 1:**
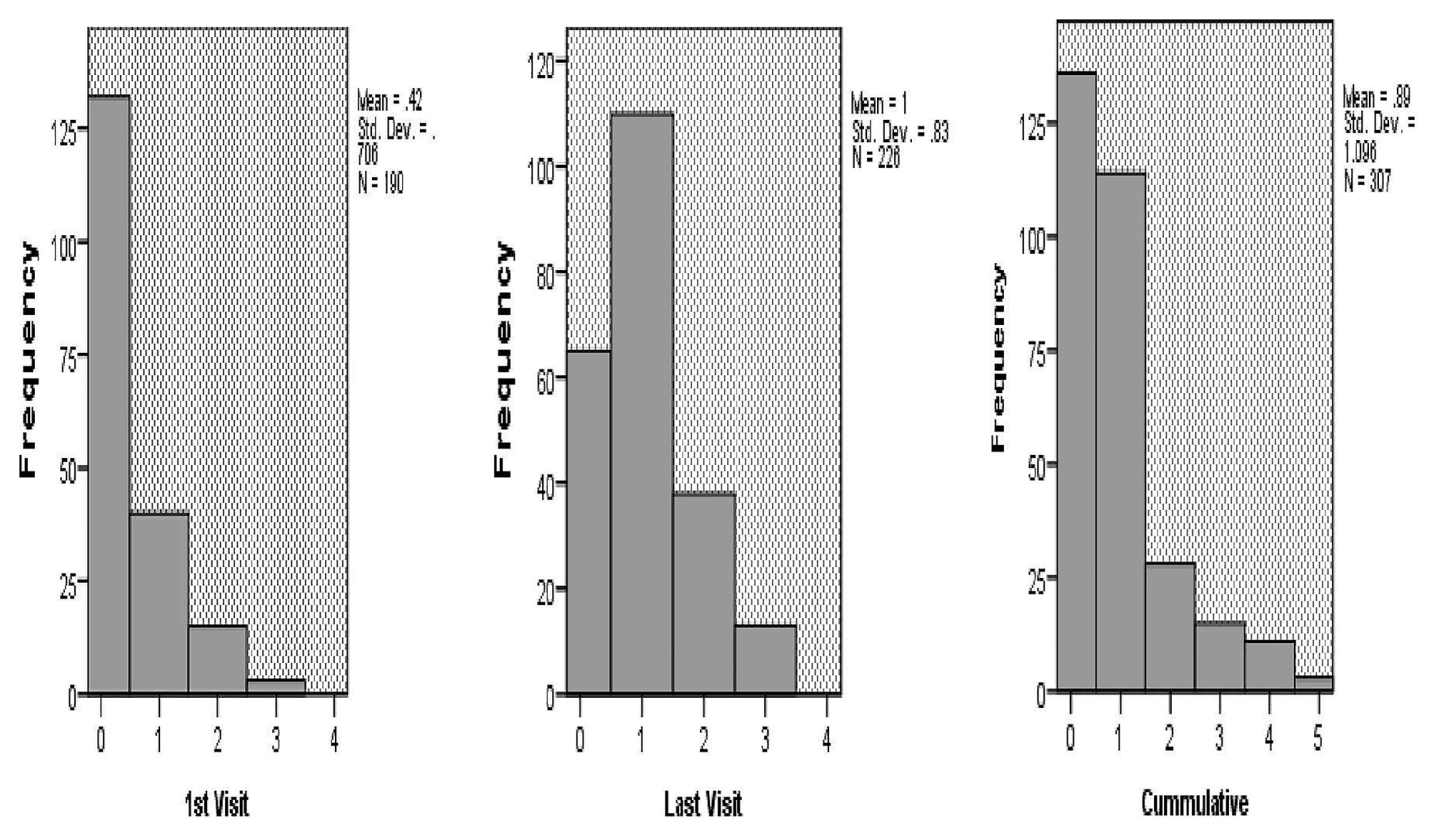
Frequency of modern contraceptive counseling among postnatal women in Arzi zone, 2022. **Key:** A category of 0 represents women who received no counseling regarding modern contraceptive at all during the contacts they had with the health professional at healthy facilities; 1 received counseling only once, 2 received counseling twice, 3 received counseling three times, 4 received counseling four times

Of 190 postnatal women who had interaction with health professionals at a health facility in the first week following the current birth, 132 (69.5%) received no advice regarding modern contraception at all, 114 (27.3%) received counseling once, 15 (7.9%) received counseling twice, and 3 (1.6%) received counseling three times or more. On the other hand, of 226 women who had contacts with health professionals at a health facility between the 8th and 42nd day, 65 (28.8%) received no advice at all, 110 (48.7%) received advice once, 38 (16.8%) counseled twice, and 13 (5.8%) received counseling three times or more. In total, 247 (59.1%) women received no counseling at all, 114 (27.3%) received counseling once, 38(9.1%) received counseling twice, and 19 (4.5%) received counseling three times or more (Fig. 1).

### Modern contraceptive uptake during postanatal period after birth

**Fig. 2:**
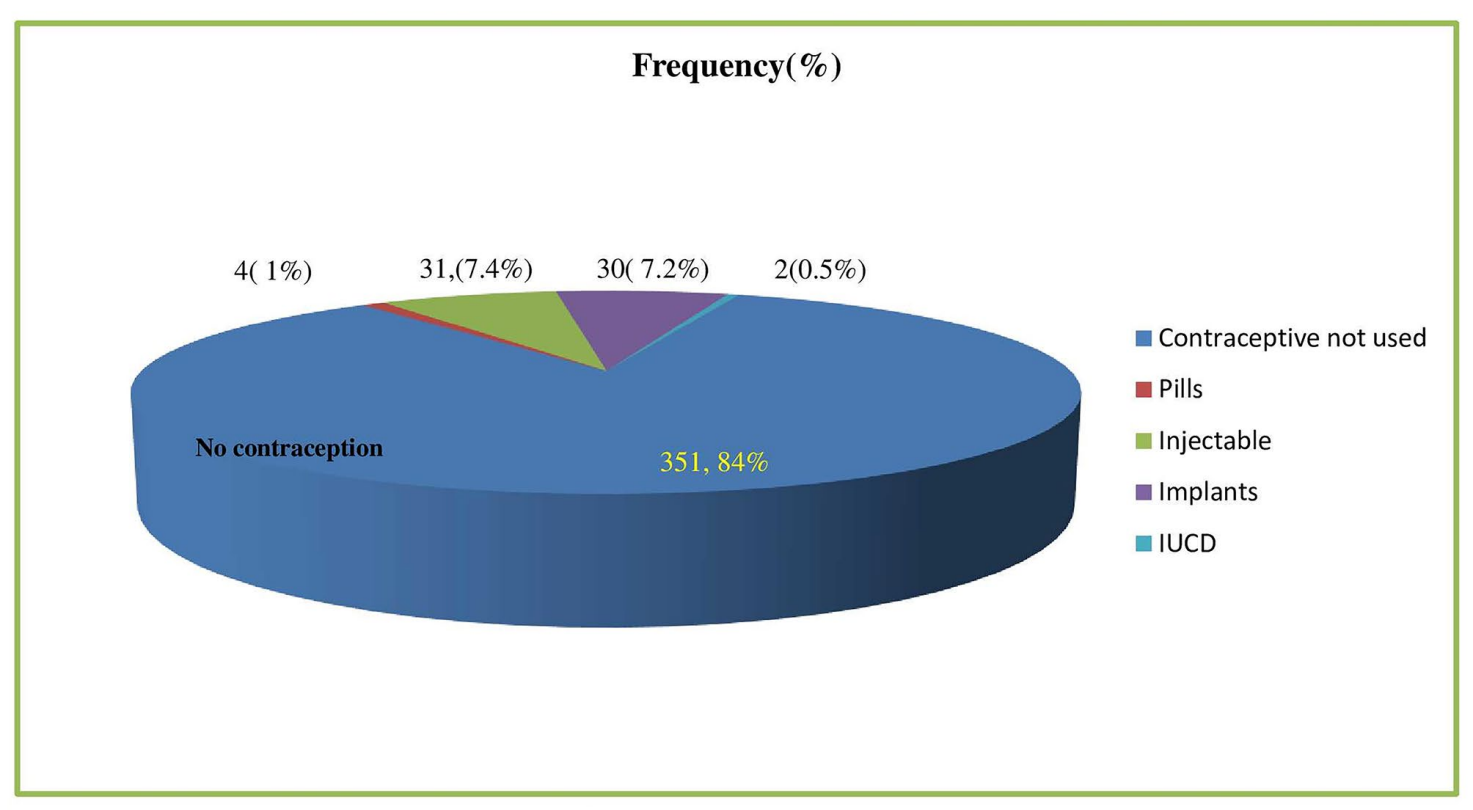
Types of contraceptive used by postnatal women in Arzi zone, 2022

At the time of the last interview, 67 postnatal women (16.0%; 95% CI: 12.50–19.50%) were using a modern form of contraception. The most commonly utilized methods of contraception were injections (31, 46.3%) and implants (30, 44.8%). Intrauterine contraceptive devices (IUCDs) usage was only 2 (3%) (Fig. 2).

### Reported reasons for not starting contraceptives

**Fig. 3:**
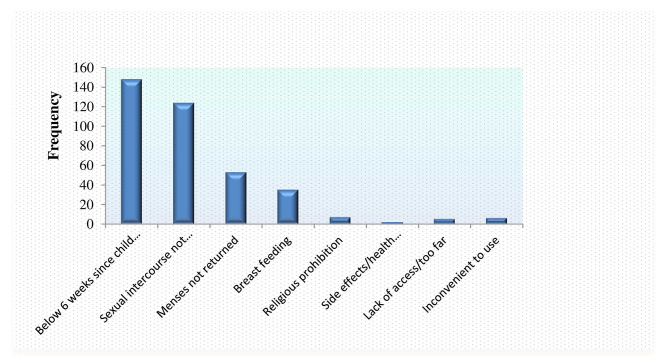
Reported reasons for not starting contraceptive among postnatal women in Arzi zone, 2022

The reasons given by women who did not start utilizing contraception after the most recent birth varied: 110 (31.3%) reported they had not yet resumed sexual activity, 69 (19.7%) were breastfeeding, 60 (17.1%) were less than six weeks postpartum, and 46 (13.1%) reported that their menses had not yet returned (Fig. 3).

### Factors associated with modern contraceptive uptake during postnatal period

Table 4 displays the adjusted relative risk (aRR) among contacts with health professionals where contraceptives were discussed or not. Postnatal women who had contacts with health professionals at a health facility and counseling about contraceptives were 3.40 times more likely to begin contraception than their counterparts (aRR = 3.40, 95% CI: 1.16–5.99). In the model, we included additional variables to control for any potential confounding influences on our main predictor. Place of residence, knowledge of whether they can become pregnant before menses return, menses returning after child birth, sexual activity beginning after childbirth, desire to have more children, and women’s knowledge of the recommended Healthy Timing and Spacing of Pregnancy (HTSP) were all significantly associated with contraceptive use during the first six weeks following childbirth (See Table 4).

**Table 4:** Factors associated to modern contraceptive among postnatal women in Arsi Zone, 2022

*Variables*	*Contraceptive use*		*cRR(95% CI)*	*ajRR (95% CI)*	*P-Value*
	**Yes**	**No**			
**Age**					
< 20	27	42	0.14(0.03–0.65)	0.19(0.02–1.61)	0.127
20–29	112	138	1.15(0.62–2.11)	1.09(0.39–3.06)	0.874
>=30	32	67	1.00	1.00	
**Residence**					
Rural	53	241	1.73(0.92–3.25)	0.25(0.07–0.88)*	0.031
Urban	14	110	1.00	1.00	
**Women Educational level**					
Cannot Read and Write	20	32	4.03(1.64–9.88)	1.03(0.13–8.06)	0.981
Can Read and Write	7	34	1.33(0.45–3.89)	5.51(0.51–59.88)	0.161
Primary education	31	227	0.88(0.40–1.95)	2.13(0.51–8.90)	0.298
Secondary and above	9	58	1.00	1.00	
**Husband Educational level**					
Cannot Read and Write	5	12	2.69(0.83–8.74)	0.50(0.05–5.52)	0.572
Can Read and Write	21	34	3.99(1.85–8.62)	3.78(0.55–25.85)	0.175
Primary education	26	208	0.81(0.41–1.60)	2.39(0.64–8.94)	0.196
Secondary and above	15	97	1.00	1.00	
**They believe they can be pregnant before menses return**					
Yes	63	186	13.97(4.98–39.22)	24.91(5.66–42.90)*	0.001
No	4	165	1.00	1.00	
**Menses Returned**					
Yes	44	39	15.30(8.36–18.01)	6.75(2.11–21.57)*	0.001
No	23	312	1.00	1.00	
**Sexual intercourse resumed**					
Yes	59	128	12.85(5.95–27.74)	3.98(1.26–12.57)*	0.019
No	8	223	1.00	1.00	
**Desire to have more children**					
Yes	2	45	0.21(0.05–0.88)	0.03(0.01–0.55)*	0.019
No	65	306	1.00	1.00	
**Desire to wait some time before the next pregnancy**					
Yes	5	63	0.37(0.14–0.95)	3.95(0.78–20.12)	0.098
No	62	288	1.00	1.00	
**Contacts with health professionals at a Health facility**					
Yes	65	161	38.35(9.25-159.01)	16.54(2.96–92.31)*	0.001
No	2	190	1.00	1.00	
**Number contacts with health professionals at a Health facility**					
One	21	93	13.90(4.81–40.18)	0.76(0.28–2.20)	0.781
Two and above	25	32	28.40(9.52–84.66)	0.59(0.23–5.61)	0.892
No HS contact	21	226	1.00	1.00	
**Contacts with health professionals where contraceptives were counseled**					
Yes	21	226	32.47(12.67–83.14)	3.40(1.16–5.99)*	0.042
No	46	125	1.00	1.00	
**Women’s Knowledge about the recommended HTSP**					
> 24 months	38	81	14.78(5.08–42.97)	17.50(18.50-61.93)*	0.001
18 months-24 months	25	144	5.47(1.85–16.14	12.30(2.94–23.45)*	0.001
< 18 months	4	126	1.00	1.00	

*statistically significant at P value < 0.05

## Discussion

A total of 418 postnatal women were enrolled in the study and interviewed twice: the first time during the first seven days following the current birth, and the second time within a week after the 42nd day of the current birth. Of the total number of postnatal women enrolled in the study, 37 (8.9%) had just begun using contraceptives at the time of the first interview, and 67 (16.0%) were doing so at the time of the last interview (within a week after the 42nd day of the current birth). This shows that although a sizable number of postnatal women had interactions with the healthcare facility, particularly in primary health care settings, very few of them received contraceptive counseling and very few actually started using contraceptives during this crucial period. Only 3% of the women were using Postpartum Intrauterine contraceptive devices (PPIUCDs), whereas 46.3% and 44.8% of the women started using injectables and implants, respectively. This was also reported in earlier researches done in Ethiopia [6, 9].

The uptake rate of modern contraceptives in this study (16%; 95% CI: 12.5–19.5%) is comparable to findings in the earlier prospective follow-up studies carried out in Bahir Dar City Administration, Northwest Ethiopia, which reported 19.1% of the contraceptive uptake rate during the first 6 weeks after childbirth [21]. However, it is lower than similar studies carried out in the Burie District, Ethiopia (20.7%) [22], the North Shoa, Ethiopia (21.3%) [23], the Gamo Zone, Southern Ethiopia (35.6%) [24], and the National level Survey in Ethiopia (27%) [25]. The differences in study design, study setting, and study population may be the reasons for the discrepancies between our results and those of studies with contraceptive use rates higher than our findings. In our study, we used a prospective cohort study design, while all the compared studies were cross-sectional studies. On the other hand, our study was community-based, whereas all the compared studies that reported a higher contraceptive use rate were facility-based, in which women who attended the health facilities may have had a better opportunity to start the contraceptive. The participants in our study were postnatal women (women who had given birth within 42 days after child birth), as opposed to some comparative studies that used postpartum women (women who had given birth within 12 months after child birth), who might have had more opportunities to start using contraceptives than postnatal women. Our study’s findings, however, are greater than the study carried out in Kebribeyah, Eastern Ethiopia, which reported 12.3% of women used contraceptives during the postpartum period [26]. The compared study was conducted in the Somali Region, the region with the lowest percentage of contraceptive prevalence rate (CPR) in Ethiopia [14], which could be the explanation for the lower contractive uptake rate compared to our result. From these comparisons in general, we can see that postnatal women in the Arsi zoneuse contraceptives at a lower rate. This may call for wise interventions to raise the rate at which contraceptives are being used in the area.

The primary factor that we intended to study in relation to contraceptive uptake throughout the postnatal period was contacts with health professionals at a health facility where contraceptives were discussed. Modern contraception was 3.40 times more likely to be started by postnatal women who had contacts with health professionals and were also counseled about contraception than by their counterparts (aRR = 3.40, 95% CI: 1.16–5.99). This finding is supported by previously conducted studies in different parts of Ethiopia: a prospective follow up study conducted in Bahir Dar City Administration, Northwest Ethiopia [21], a cross-sectional study in Gamo Zone, Southern Ethiopia [24], national surveys in Ethiopia [25], a cross-sectional community based survey conducted in Kebribeyah Town, Somali Region, Eastern Ethiopia [26], a community based cross-sectional study conducted in northern Ethiopia [27], and a facility-based cross-sectional study conducted in Addis Ababa, Ethiopia [28]. This implies that postnatal family planning needs to be discussed at every opportunity so that a woman can begin her preferred contraceptive methods as soon as possible after childbirth [29]. Women coming to health facilities for Maternal, Neonatal, and Child Health (MNCH) services during the postnatal period should be given counseling about contraceptive use so that their contraceptive needs during this critical time can be met. As a result, the immediate postpartum family planning (IPPFP) uptake rate will be improved in the study area.

In our study, mere health professional contact was associated with contraception uptake during the postnatal period. Women who had contacts with health professionals at a health facility after childbirth were more likely to use a modern contraception in the first six weeks following childbirth, even though the confidence interval was not precise (aRR = 16.54, 95% CI: 2.96–92.31). This finding was in line with the earlier studies conducted in Bahir Dar City Administration, Northwest Ethiopia [21], a community-based cross-sectional study conducted in northern Ethiopia [27], a community-based cross-sectional study conducted in Dubti Town, a Pastoral Community, of Afar Region Northeast, Ethiopia [30], and another study carried out in Ethiopia [31]. This may be due to the fact that women who have had a chance to contact the health professionals at the health facility for MNCH service may have an opportunity to access information about contraception and, as a result, may uptake it soon after childbirth. This may imply the importance of encouraging women to visit health facilities after childbirth.

In addition to the main predictor, other reproductive characteristics were associated with contraceptive uptake during the postnatal period. Women were more likely to use a modern contraceptive if they knew they could become pregnant before menses return (aRR = 24.91, 95% CI: 5.66–42.90), if her menses had returned (aRR = 6.75, 95% CI: 2.11–21.57), and if she had resumed sexual intercourse following childbirth (aRR = 3.98, 95% CI: 1.26–12.57). The women’s choice to utilize contraceptives throughout the postpartum period was also influenced by their knowledge of the recommended healthy timing and spacing of pregnancies (HTSP). Contraceptives were used more frequently during the postpartum period among those who said the HTSP should be beyond 24 months (aRR = 17.50, 95% CI: 18.50-61.93) and between 18 and 24 months (aRR = 12.30, 95% CI: 2.94–23.45) compared to those who believed it should be under 18 months. These results are consistent with those of earlier studies carried out in Burie District, Amhara Region, Ethiopia [22], a community-based cross-sectional study carried out in northern Ethiopia [27], a cross-sectional study carried out in Addis Abeba, Ethiopia [28], and another community-based cross-sectional study carried out in Arba Minch town, Southern Ethiopia [32]. These findings, which are related to women’s family planning literacy, would suggest that women can start family planning right away after giving birth if they have the right information about it. This may be the justification for the National Guideline for Family Planning Services in Ethiopia’s emphasis on increasing family planning knowledge [33].

Women’s place of residence was also associated with the use of contraception in the six weeks following childbirth. When compared to urban women, rural residents had a lower likelihood of using contraceptives (aRR = 0.25, 94% CI: 0.07–0.88). This finding is similar with EDHS based data analysis in Ethiopia [31]. This may be due to the fact that, women living in the urban area may have better access to the family planning information and services compared to women living in the rural area.

The study’s strength was the prospective cohort design that was used. There are certain limitations in our study that are worth reporting. Some of the information relied on the past memories of the interviewed women, which could lead to recall biases. We obtained data through the self-report of the interviewed women. As a result, the data accuracy might not be at a level that can be obtained objectively. This may also result in social desirability bias. Nevertheless, women were thoroughly informed about the value of accurate information through explaining the purpose of the study and also ensuring privacy, anonymity, and confidentiality. The precision of our estimation may have been impacted by the noticeably broader confidence interval we observe for one of the predictors (contacts with health professionals). This is attributed to the extremely low frequency (which is 2) that we found in the cross-tabulation of the two variables: contraceptive uptake and contacts with health professionals at a health facility. Yet, it is valid that there was a statistically significant association between contraception uptake and contacts with health professionals at a health facility.

## Conclusion and recommendations

The modern contraceptive uptake rate among postnatal women in the study area was found to be low. Despite the postnatal women’s desire to begin using modern contraceptives to space pregnancies, they could not do that and were therefore at risk of mistimed pregnancy. Contact with health professionals where contraceptives are discussed was the main predictor in this study. However, the majority of women did not receive counseling about contraceptives during their contacts with health professionals at a health facility. Mere contact with health professionals at a health facility was also associated with contraception uptake during the postnatal period. In addition, knowledge of health timing and spacing for pregnancy, knowing they can become pregnant before menses return, menses return, and resuming sexual intercourse after child birth were reproductive characteristics associated with contractive use. Age and place of residence of women also showed association with contraceptive uptake during the postnatal period.

We recommend Arsi zonal health office and district health offices routinely provide sensitization workshops for health workers in the district focusing on modern contraceptive counseling, especially to postnatal women, at community level to reach all eligible women in the general population. Regular monitoring and supportive supervisions on proper counseling for eligible women in all health facilities during the postnatal period should also get attention. In order to reduce the proportion of women with short inter-pregnancy intervals, we also advise the healthcare professionals and health extension workers working in the Arsi Zone health facilities to strengthen contraceptive counseling in postnatal health services. Moreover, we strongly recommend that health centers in the districts integrate contraceptive counseling into all MNCH services like delivery service, PNC, and EPI.

## Data Availability

Data sets supporting the presented findings were incorporated into the manuscript and are available from the corresponding author on reasonable request.

## Abbreviation

ANC: Antenatal care
AOR: Adjusted Odds Ratio
CI: Confidence Interval
FGD: Focus Group Discussion
FMOH: Federal Ministry of Health
FP: Family planning
HEW: Health Extension Workers
HTSP: Healthy Timing and Spacing of Pregnancy
IUD: Intra uterine Device
LAM: Lactation Amenorrhea Method
MNCH: Maternal, newborn and child health
OR: Odds Ratio
PHCU: Primary Health Care Unit
PNC: Postnatal care
PPFP: Postpartum Family Planning
PP: Postpartum Period
PPIUD: Postpartum intrauterine device
SPSS: Statistical Package for Social Sciences
WHO: World Health Organization
